# A multi-institutional partnership catalyzing the commercialization of medical devices and biotechnology products

**DOI:** 10.1017/cts.2021.779

**Published:** 2021-04-08

**Authors:** Nathaniel Hafer, Bryan Buchholz, Denise Dunlap, Brennan Fournier, Scott Latham, Mary Ann Picard, Steven Tello, Laura Gibson, Craig M. Lilly, David D. McManus

**Affiliations:** 1 UMass Center for Clinical and Translational Science, Worcester, MA, USA; 2 Massachusetts Medical Device Development Center, Lowell, MA, USA; 3 Program in Molecular Medicine, UMass Medical School, Worcester, MA, USA; 4 Department of Biomedical Engineering, UMass Lowell, Lowell, MA, USA; 5 Manning School of Business, UMass Lowell, Lowell, MA, USA; 6 Department of Medicine, UMass Medical School, Worcester, MA, USA; 7 Department of Pediatrics, UMass Medical School, Worcester, MA, USA; 8 Department of Anesthesiology and Perioperative Medicine, UMass Medical School, Worcester, MA, USA; 9 Department of Surgery, UMass Medical School, Worcester, MA, USA; 10 Department of Population and Quantitative Health Sciences, UMass Medical School, Worcester, MA, USA

**Keywords:** Medical devices, biotechnology, entrepreneurship, innovation, education

## Abstract

The commercialization of medical devices and biotechnology products is characterized by high failure rates and long development lead times particularly among start-up enterprises. To increase the success rate of these high-risk ventures, the University of Massachusetts Lowell (UML) and University of Massachusetts Medical School (UMMS) partnered to create key academic support centers with programs to accelerate entrepreneurship and innovation in this industry. In 2008, UML and UMMS founded the Massachusetts Medical Device Development Center (M2D2), which is a business and technology incubator that provides business planning, product prototyping, laboratory services, access to clinical testing, and ecosystem networking to medical device and biotech start-up firms. M2D2 has three physical locations that encompass approximately 40,000 square feet. Recently, M2D2 leveraged these resources to expand into new areas such as health security, point of care technologies for heart, lung, blood, and sleep disorders, and rapid diagnostics to detect SARS-CoV-2. Since its inception, M2D2 has vetted approximately 260 medical device and biotech start-up companies for inclusion in its programs and provided active support to more than 80 firms. This manuscript describes how two UMass campuses leveraged institutional, state, and Federal resources to create a thriving entrepreneurial environment for medical device and biotech companies.

## Introduction

The commercialization of innovative biomedical technologies is complex, time and resource intensive, and fraught with high failure rates. The University of Massachusetts Medical School (UMMS) and University of Massachusetts Lowell (UML) have a long-standing partnership to address these challenges and help innovators, especially start-up companies, commercialize their technologies. This manuscript describes the innovative programs that UMMS and UML have jointly developed as a guide to other academic institutions and companies wanting to design, expand, and/or improve their entrepreneurial ecosystems.

Biotechnology incubators are designed to help nurture new companies and limit costs related to capital equipment and administrative services. Incubators can operate as for-profit or not-for-profit; some take an equity stake in their resident companies and others do not. Incubators can also be grouped according to their affiliation (or lack of affiliation) with an academic center, local business development effort, or specific type of industry focus [1,2].

The Massachusetts Medical Device Development Center (M2D2) is a technology and business incubator within the medical device and biotech industry. Established in 2008, the mission of M2D2 is to provide a lifeline for the state’s emerging medical device companies, offering inventors and executives easy, affordable, and coordinated access to world-class researchers and resources at the UML and UMMS campuses. These efforts help entrepreneurs bridge the product development “valley of death” – that is the gap between academic research and commercial product launch [3,4]. M2D2 combines the engineering and business expertise of UML faculty with the clinical expertise of UMMS. Program staff has provided grant writing guidance, business development advice, and new company creation assistance. The center also forges connections to University faculty, students, and core research facilities. Access to physical incubator and office space, prototyping equipment, and clinicians for assistance with clinical studies are additional resources.

Over the past 10 years, M2D2 has helped over 80 start-up companies advance the development of their medical devices and biotech innovations. There are currently 43 companies that rent affordable laboratory space, close to but outside of the Kendall Square area of Boston (i.e., often referred to as the center of the nation’s biotechnology industry) and dozens more that are virtual tenants (i.e., they use M2D2 services but have a limited physical presence in the incubator). Due to the success of M2D2’s programs, 18 companies have graduated due either to acquisition or to the need to expand beyond the incubator space; only 6 companies have failed to date.

## Leadership and Governance

**Fig. 1.**
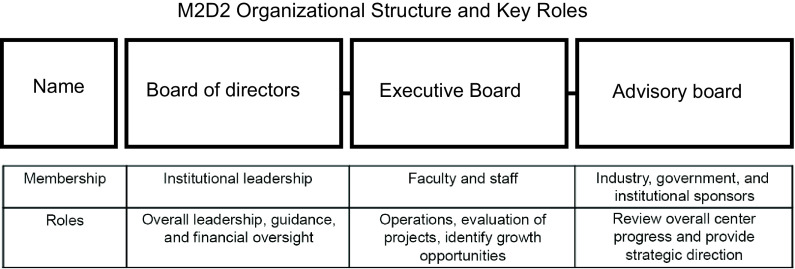
The Massachusetts Medical Device Development Center (M2D2) is led by three different boards: a board of directors, an Executive Board, and an advisory board.

Since M2D2 is a joint effort it draws from both institutions and leverages their complementary expertise. It is organized as a center within the institutional structure of each campus. UML draws from its strong engineering, science, and business tradition, while UMMS brings unparalleled medical expertise. M2D2 has a board of directors, an Executive Board, and an advisory board (Fig. 1). The board of directors is made up of leaders from each campus who provide overall leadership, guidance, and financial oversight of the center. The Executive Board meets once every 2 weeks. At these meetings, the Executive Board discusses operational plans, evaluates the on-going progress of medical device and biotech entrepreneurs, and identifies target organizations. Executive Board members come from both campuses and have expertise in nursing, technology transfer, engineering, business, clinical disciplines, health sciences, environmental health and safety, advancement, and media relations. The advisory board’s primary function is to assist with the evaluation of the innovative potential of early-stage ventures. Advisory board members meet on a bi-annual basis to offer their assessment of target companies for investment. This board is made up of senior executives from M2D2 sponsor companies such as Johnson & Johnson, Boston Scientific, and Amgen. Other business sponsors include representatives from the legal, prototyping, manufacturing, and regulatory industries. University sponsors, including the five-campus UMass Center for Clinical and Translational Science (UMCCTS), also sit on this board and provide significant institutional support.

Prospective applicants are accepted into the M2D2 community through the following process. First, the interested company completes an application form and schedules a presentation at a bi-weekly Executive Board meeting. During the meeting, the company presents a typical “business pitch deck,” describing the unmet need, the proposed solution, funds raised to date, intellectual property secured, the team and their expertise, and specific ways that the company plans to interact with the incubator. Next the Executive Board votes to accept or reject the applicant based on all these factors. Accepted companies complete a standard lease agreement and lab safety training before moving in. Over the past year, occupancy rate across all three sites has averaged between 85% and 90%. Of the approximately 40 companies in residence in February 2021, 10%–15% are UMass spinouts, 30%–35% are academic spinouts, and 20%–25% were originally based out of state. Companies that are deemed too early to move in receive individual feedback about their project and remain eligible to participate in all of our educational programming, office hours, networking, and competitions.

## Resources and Services

M2D2 offers various business and technical services intended to increase the probability of early-stage success. M2D2’s services broadly fall into one of the following categories: physical space; technical and engineering assistance; clinical and medical assessment, business development assistance; and networking and outreach (Table 1).

**Table 1.** Massachusetts Medical Device Development Center (M2D2) resources are grouped into five broad areas of support. Resident companies pay rent or membership fees to access these resources

Type of assistance	Examples
Physical space	Wet lab space
	Core resources
	Office and shared space
Technical and engineering assessment	Materials selection
	Product design and prototyping
	Product manufacturing including injection molding, extrusion, blow molding, and material testing
	Friction characterization, failure analysis, advanced materials characterization, and occupational biomechanics
Clinical and medical assessment	Expert review and feedback in areas such as cardiology, orthopedics, surgery, and diabetes
	Access to patient populations
	Regulatory support
Business development	Business plan development
	Feasibility and business scan development
	Marketing plan development
	Partner identification
Networking and outreach	Educational programs
	Mentoring
	Pitch events and networking with banks, angel investors, venture capitalists, and industry partners

### Physical Space

Physical space serves as an anchor for all these activities. In 2011, an initial investment of $4 million from the Massachusetts Life Sciences Center (MLSC), a state agency, supported the construction of the Wannalancit Mill space in Lowell. This 24,000 square foot project created 6 private laboratories with shared office and conference space. In 2015, UML again partnered with MLSC to develop a second incubator site at 110 Canal Street in Lowell. This $4 million project created 48 benches in an 11,000 sq ft open-concept lab space along with shared office space, shared equipment, BSL2 capabilities, and conference space. Most recently, M2D2 Worcester opened at the UMMS. This 1,500 sq ft space includes shared equipment, office, and conference room space along with 10 lab benches in an open-concept laboratory environment. M2D2 has critical lab equipment to support early product development and importantly, as M2D2 increases capacity, it also increases economies of scale related to equipment purchase, use, and maintenance. In the past year, over $150,000 has been invested in equipment and upgrades. Resident companies are polled annually to see what kinds of shared equipment and resources are in demand, and M2D2 leadership acquires new instruments based on these responses. Access to common equipment within the M2D2 space is included as part of rent. Equipment and core resources that are not immediately contained within the lab space can be made available to companies with proper training and regulatory approvals [5,6]. Resident companies receive preferred rates at these cores and also qualify for a state-supported core resource voucher program [7,8]. Biosafety cabinets, environmental rooms, and other resources within the larger UMass environment are also available.

### Engagement

As an incubator operated by a major research university, one of the most valuable services that M2D2 provides are connections to the UMass innovation ecosystem. The diverse representation of Executive Board, which includes leaders of the UMCCTS and campus technology transfer offices, facilities connections to people and resources across UMass. M2D2 staff frequently introduce companies to UMass faculty for clinical insights, engineering expertise, and business advice. M2D2 Executive Board members often make the initial introduction and additional connections are made according to the needs of the company. The vast majority of faculty are very willing to meet with these start-ups. Faculty have expressed interest in learning about emerging trends/technology and are motivated to help early-stage entrepreneurs. Interactions can be relatively brief and informal or develop into multi-year sponsored research programs. UMass students are another major resource for M2D2 companies. In collaboration with the MLSC Internship Challenge [9], companies gain access to students eager for real-world experience. At the end of the internship, the state MLSC program reimburses the company for the cost of the student’s stipend. Overall, M2D2 has placed over 120 students in our client companies and over 30 have developed into valued part- or full-time employees.

### Industry Partners

Industry sponsors are an essential element of the entrepreneurial environment. In addition to providing financial support to M2D2 operations, sponsors support emerging companies through access to experienced entrepreneurs and large company expertise in deal-making, key technologies, product development, and commercialization. Some support comes in the form of educational programming through symposia and workshops. Programming is developed collaboratively between M2D2 leadership and industry sponsors based on feedback and survey responses from our membership. In 2020, M2D2 hosted 26 events with 1,870 attendees on topics including fund raising, mergers and acquisitions, intellectual property, and employment law. Additionally, 71 office hour sessions were held between sponsors and 387 resident attendees. Informal networking at events and through office hours are other ways new innovators learn valuable lessons about starting successful companies. Industry sponsors frequently remark that residents are highly motivated and responsive to input. These educational interactions often lead to long-term business relationships that benefit both the mentor and mentee.

### Funding and Networking

Annual pitch events are another way M2D2 partners with industry sponsors to provide start-ups the exposure and access to capital that is critical to bring technologies to market. M2D2’s signature event is the “M2D2 $200K Challenge,” an annual competition designed to identify and reward up-and-coming companies in the medical device and biotechnology industries. Each year entrepreneurs are invited to pitch their technologies to a panel of experts selected from our industry sponsors. The competition has grown from 15 local applicants in 2012 to over 200 applicants from around the world in 2020. After an initial round of reviews conducted by the M2D2 Executive Board, selected finalists present their technology at a pitch and networking event to win a share of $200,000 of sponsor-provided, in-kind services. These services include laboratory space along with engineering, product development, legal, regulatory, clinical, and business services.

Another pitch event is the “Barracuda Bowl,” a panel-style event modeled after a popular television show. Several entrepreneurs pitch their venture to a panel of investors and face questions regarding their technology, current stage of development, and financial projections. Over the past 6 years, 40 different entrepreneurs have presented their technologies to 42 investors in front of over 800 attendees.

These events have grown in popularity through a combination of factors: company word of mouth; advertising in trade publications and meetings; outreach via M2D2 sponsor and funding networks (see below); and partnerships with international business organizations. M2D2 works closely with the UMass tech transfer office on each campus to identify and nurture technologies that may be a good fit for the incubator. Members of the tech transfer office participate on the Executive Board. M2D2 does not take an equity stake in any company nor does it make any claim on IP developed in our facilities.

## Federally Supported Research Centers

Over the first 10 years of its’ existence, M2D2 developed through the hard work of the founding directors, board members, institutional champions on each campus including the UMCCTS, and significant support from state and internal resources. As the program has matured and demonstrated value to start-ups and established companies, several Federally funded, peer reviewed centers have been established.

### Center for Advancing Point of Care Technologies

**Fig. 2.**
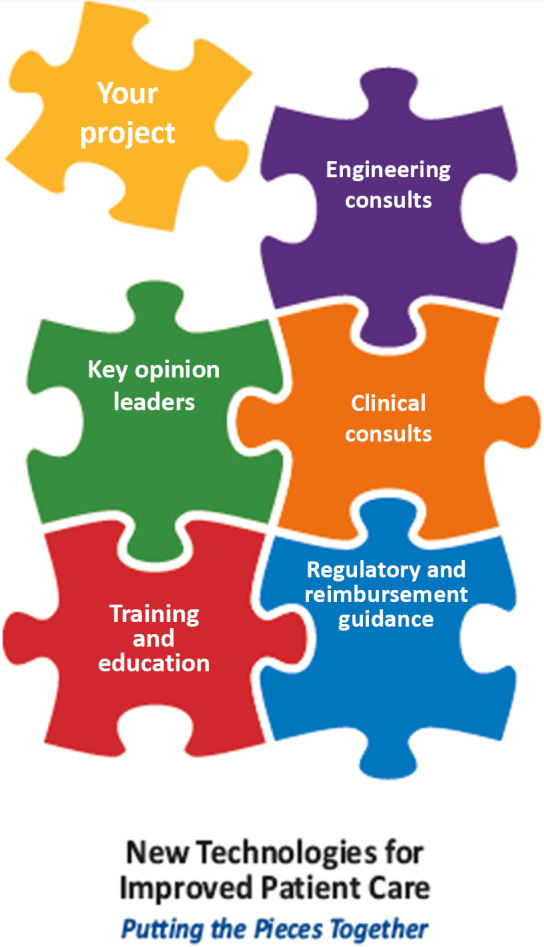
CAPCaT resources. The Center for Advancing Point of Care Technologies (CAPCaT) in heart, lung, blood, and sleep disorders combines the expertise of the Massachusetts Medical Device Development Center (M2D2) with National Institutes of Health (NIH) resources to accelerate the commercialization of point of care technologies.

In 2018, UMMS and UML received a 5-year, $7.9 million award from the National Institutes of Health (NIH) to establish the Center for Advancing Point of Care Technologies (CAPCaT) in heart, lung, blood, and sleep disorders. CAPCaT is one of five centers developing a pipeline of technologies with commercialization potential called the Point of Care Technology Research Network (POCTRN) [10]. Innovations aim to help patients with these health concerns better manage their well-being wherever they are, seeking to reduce in-patient hospital stays and improve quality of life. While a major function of CAPCaT is to solicit and fund pilot projects, awardees are considered M2D2 resident companies and are therefore eligible to receive all the services and resources described above (Fig. 2). In addition, these innovators receive added benefits such as participation in our annual innovator showcase, the Biotech East program, and the I-Corps short course.

The innovator showcase event is designed to introduce our pilot grant awardees to potential large strategic investors and additional funding opportunities. Further, the event is offered to the public to provide exposure to the awardees and to allow attendees, made up mainly of entrepreneurs, to learn directly from experts about best practices when seeking large strategic investments. Representatives from the NIH’s small business programs are also included to share information about funding opportunities and best practices for Small Business Innovation Research and Small Business Technology Transfer applications. Our first event was held virtually in June 2020 due to the COVID-19 pandemic. Over 300 participants registered from 32 states and 7 countries. Sponsors included MPR Associates, Johnson & Johnson, Siemens Healthineers, Philips Ventures, Boston Scientific, Amgen Ventures, and NIH.

The Biotech East program was founded in 2017 and is a joint collaboration between UML, the American Society of Cell Biology, and Keck Graduate Institute (KGI) of the Claremont Colleges. The Biotech East program builds on the model developed in 2014 at KGI. The program offers a week-long, hands-on course to help US and international advanced graduate students, PhD students, and postdoctoral fellows transition to careers in biotech, medtech, or pharma [11]. Programming teaches students the business side of science, professional development, networking skills, and interdisciplinary skills through MBA-style cases, keynote speakers, panels, and a team project. M2D2/CAPCaT is featured prominently in lectures, panels, and tours throughout the program, which draws students from top universities, such as the Massachusetts Institute of Technology, Harvard, Yale University, the University of Pennsylvania, and the University of Michigan. The program has gained an international reputation with students traveling from as far as Australia, Brazil, Austria, Israel, and India to attend the program. Currently, nearly 70% of attendees that have attended the program have gone onto non-academic careers in industry, regulatory affairs, or tech transfer firms.

The I-Corps short course program, a joint CAPCaT/UMCCTS initiative, is designed to support the commercialization of biomedical research by providing training in state-of-the-art methods and strategic guidance to faculty, staff, and students during the early phases of technology development [12]. The canonical I-Corps course offered by the NSF and NIH is intense, requiring 30+ hours of work per week for 7–8 weeks [13–15]. Many small companies and academic inventors cannot afford that time commitment, especially in the early stages of development. CAPCaT leadership, along with colleagues at eight other CTSA sites, are teaching an I-Corps short course curriculum specifically targeting medical product innovations, including point of care technologies. We plan to develop, evaluate, and disseminate a point of care technology module for the short course. The goal of this short course is to prepare participants to focus their R&D efforts beyond the laboratory, broaden the impact of their efforts, and enhance their capability to translate discoveries into commercially successful products that improve human health. A key element is to ensure that participants understand the patient care process – e.g., how innovations impact the current health care delivery model, how mobile devices create communication and POC monitoring opportunities, and how to distinguish product users from beneficiaries and purchasers. The I-Corps program is offered annually for CAPCaT awardees, and all are expected to take this training as part of their award. Teams that achieve milestones and are chosen for further development will be encouraged to attend the full NSF/NIH I-Corps program offered at the regional or national level.

### Rapid Acceleration of Diagnostics

In response to the COVID-19 pandemic, the NIH established the Rapid Acceleration of Diagnostics (RADx) initiative to speed innovation in the development, commercialization, and implementation of technologies to detect SARS-CoV-2 nucleic acid and antigen, with the goal of making millions of tests per week available to Americans. RADx is a nationwide call for start-ups, established companies, and academic scientists to bring forth their novel, innovative ideas for new coronavirus testing methods. Those selected received funding and wrap-around services to move the approaches and strategies forward. As a member of POCTRN, UML and UMMS were selected by NIH leadership to play a key role in this effort. These activities were generally divided along the historical strengths of each campus. UML worked with nine RADx applicant companies to conduct preclinical stage experiments such as limit of detection studies, feasibility studies to achieve Emergency Use Authorization, system performance studies, cross-reactivity and analytical specificity studies, and to provide usability feedback. Clinical research activities included a biorepository and studies of device usability, feasibility, and comfort. UMMS was selected to lead the clinical studies core, providing expertise in study design, management, data analysis, regulatory affairs, and logistics. Within 2 months of funding, the clinical studies core opened their first study; within another 4 months, the program had opened 5 multi-site studies and enrolled over 1,000 participants. In all cases, UMass resources were provided to innovators as an additional service to their RADx awards. We predict that without these services, many small companies that participated in the RADx program would have had their project timelines delayed by the regulatory, preclinical, and clinical development process barriers that we identified and resolved.

### Biomedical Advanced Research and Development Authority DRIVe

The Biomedical Advanced Research and Development Authority (BARDA) is an office within the Department of Health and Human Services responsible for the procurement and development of medical countermeasures. M2D2 received a 5-year, $500,000 award from BARDA’s Division of Research, Innovation, and Ventures to scout innovative solutions looking to solve our nation’s greatest healthcare problems and aid in development process of more efficient medical countermeasures [16].

M2D2 is 1 of 13 incubators/accelerators that was chosen to be part of BARDA DRIVe’s National Accelerator Network. As the only New-England based accelerator program chosen for this initiative, M2D2 has been instrumental in connecting promising health security solutions from the Boston innovation ecosystem to crucial resources needed to advance their medical technology. In 2020, M2D2 hosted several programs such as how to advance innovation in the health security space, how to get a start-up technology into large hospital systems, and how to generate exposure for healthcare solutions looking to solve unmet clinical needs and improve health worldwide. These programs reached over 750 people, showcased 9 promising innovations, and were seen in 14 countries. M2D2 has connected over 100 start-ups to BARDA’s resources, provided 7 companies with wrap-around support services, and facilitated over 11 market research calls with BARDA leadership. Areas of focus have included COVID-19, sepsis, bioterrorism, artificial intelligence, early notification of diseases, and patient tracking and monitoring tools. M2D2 supports entrepreneurs in this space by providing our incubator resources, office hours, mentorship, and business development services to prospective and current BARDA DRIVe portfolio companies.

## Conclusions

M2D2 has created a thriving entrepreneurial environment for medical device and biotechnology companies that want to commercialize their innovative products with support of a non-profit, academic partner. While early on M2D2 solely relied on support from the state, the UMass system, and industry partners, it has achieved a point of financial self-sufficiency and stability. A key lesson was to focus on a specific sector – medical devices – early on and gradually expand into other sectors. M2D2’s metrics of success are similar to other incubators: number of start-ups in residence; number and trend of graduates, scale ups, and failures; new job creation; financial support and sponsorship from partners; and the number of UMass faculty and students engaged. M2D2’s success can be attributed to several factors: strong system and state support; diligent oversight and management of resources; dedicated faculty, staff, and partners; careful and deliberate growth; and committed external partners. As M2D2 and the rest of the world eagerly await the end of the COVID-19 pandemic, all involved continue to work diligently to ensure that innovation and entrepreneurship continue to thrive in this challenging environment. Currently, M2D2 is finalizing a strategic plan that was developed with internal and external stakeholders. This plan will incorporate emerging health trends that provide unprecedented opportunities for medical device and biotech developers in the coming years including remote monitoring, virtual medicine, and digital health. This joint collaboration between UML and UMMS serves as a robust example of a high performing, university innovative ecosystem.
